# The Impact of Varying Pasture Levels on the Metabolomic Profile of Bovine Ruminal Fluid

**DOI:** 10.3390/metabo14090476

**Published:** 2024-08-28

**Authors:** Claire Connolly, Mark Timlin, Sean A. Hogan, Tom F. O’Callaghan, André Brodkorb, Michael O’Donovan, Deirdre Hennessy, Ellen Fitzpatrick, Kieran McCarthy, John P. Murphy, Lorraine Brennan

**Affiliations:** 1 UCD School of Agriculture and Food Science, Institute of Food and Health, UCD, Belfield, D04 V1W8 Dublin, Ireland; claire.connolly1@ucd.ie (C.C.); mark.timlin@teagasc.ie (M.T.); 2 UCD Conway Institute of Biomolecular and Biomedical Research, University College Dublin, D04 V1W8 Dublin, Ireland; 3 Food for Health Ireland, University College Dublin, Belfield, D04 V1W8 Dublin, Ireland; 4 Teagasc, Food Research Centre, Moorepark, Fermoy, P61 C996 Cork, Ireland; sean.a.hogan@teagasc.ie (S.A.H.); andre.brodkorb@teagasc.ie (A.B.); 5 School of Food and Nutritional Sciences, University College Cork, T12 Y337 Cork, Ireland; tom_ocallaghan@ucc.ie; 6 Teagasc, Animal and Grassland Research and Innovation Centre, Moorepark, Fermoy, P61 P302 Cork, Ireland; michael.odonovan@teagasc.ie (M.O.); deirdrehennessy@ucc.ie (D.H.); kieran.mccarthy@teagasc.ie (K.M.); johnpaul.murphy@teagasc.ie (J.P.M.); 7 School of Biological, Earth and Environmental Sciences, University College Cork, T23 N73K Cork, Ireland; 8 Teagasc, Environmental Research Centre, Johnstown Castle, Y35 Y521 Wexford, Ireland; ellen.fitzpatrick@teagasc.ie

**Keywords:** bovine ruminal fluid, nuclear magnetic resonance spectroscopy, diurnal, metabolites, pasture, total mixed ration

## Abstract

A pasture or concentrate-based dietary regime impacts a variety of factors including both ruminal health and function, and consequently milk production and quality. The objective of this study was to examine the effect of feeding differing pasture levels on the metabolite composition of bovine ruminal fluid. Ruminal fluid was obtained from rumen-cannulated spring-calving cows (N = 9, Holstein-Friesian breed, average lactation number = 5) fed one of three diets across a full lactation season. Group 1 (pasture) consumed perennial ryegrass supplemented with 5% concentrates; group 2 received a total mixed ration (TMR) diet; and group 3 received a partial mixed ration (PMR) diet which included pasture and a TMR. Samples were taken at two timepoints: morning and evening. Metabolomic analysis was performed using nuclear magnetic resonance (^1^H-NMR) spectroscopy. Statistical analysis revealed significant changes across the dietary regimes in both morning and evening samples, with distinct alterations in the metabolite composition of ruminal fluid from pasture-fed cows (FDR-adjusted *p*-value < 0.05). Acetate and butyrate were significantly higher in samples derived from a pasture-based diet whereas sugar-related metabolites were higher in concentrate-based samples. Furthermore, a distinct diurnal impact on the metabolite profile was evident. This work lays the foundation for understanding the complex interaction between dietary regime and ruminal health.

## 1. Introduction

The complex and diverse array of microorganisms present in the rumen play a critical role in both nutritional and physiological functions of the cow [1,2,3,4]. This ecosystem promotes the growth of microbes and the formation of various compounds, which are readily absorbed through the rumen walls [4,5,6]. Examples of such compounds include the short-chain fatty acids, which have a range of functions in the overall health and productivity of cows [1,7]. Many studies document the importance of various feeding regimes in relation to milk production and quality. However, in order to truly understand the relationships between the host metabolism and overall productivity, the interplay between diet and the complexities of the rumen metabolome and its overall functionality warrants investigation, not only to optimise milk production but also for the considerations of animal health and overall sustainability.

Bovine ruminal fluid is a complex biological matrix composed of inorganic ions and gases, amino acids, dicarboxylic acids, lipids such as phospholipids, short-chain fatty acids, diglycerides, triglycerides, cholesterol esters and carbohydrates [4,8,9]. In intensive feeding systems, where the cows are fed a high-concentrate diet containing 80:20 concentrate to ryegrass, there are elevated levels of alanine, leucine and glucose within the rumen metabolome [10]. Furthermore, the consumption of a grass (perennial ryegrass (Lolium perenne)) silage versus red clover (Merviot and Rozeta Trifolium pratense) silage resulted in elevated ruminal concentrations of metabolites including acetate, propionate and butyrate, again highlighting the influence of pasture regime on the metabolite composition of ruminal fluid [11]. Compounds such as acetic acid play a critical role in energy supply, with ruminal acetic acid concentrations significantly higher in total mixed ration (TMR)-fed cows [12]. Moreover, modifying barley grain concentrations within a cow’s diet results in alterations to rumen metabolite classes such as amino acids, short-chain fatty acids and other compounds [13,14]. Metabolites including acetate, valerate, methylamine and ethanol are significantly higher in ruminal fluid obtained from cows fed mixed forage diets consisting of hay and corn silage compared to corn stover [15]. However, increasing the proportion of cereals within the diet results in the perturbation of certain rumen metabolites including ethanol, putrescine and methylamines, leading to the release of harmful pro-inflammatory or toxic compounds and decreasing the ruminal pH to an acidic level, contributing to various health implications and the development of various metabolic disorders [13,14,16]. However, feeding a high (70%) forage diet, featuring grass products such as corn silage and Chinese wildrye, increased rumen pH, therefore negating various health implications associated with an acidic pH [6]. These results highlight the pivotal role which dietary regime plays on rumen ecosystem and metabolome, and subsequently on the overall health status of the animal.

Different levels of pasture feeding are employed around the world, resulting in alterations to the gross composition and nutritional profile of milk [17,18]. As the rumen plays a key role in the overall health and well-being of the cow, it is important to understand how a pasture, concentrate or hybrid partial mixed ration (PMR) diet impacts metabolite composition [12]. The PMR diet merges the traditional outdoor pasture-based system with an indoor concentrate-based diet, allowing for elevated milk yields through increased dry matter intakes, while reducing feed costs incurred by the farmer [17,18]. Previous work examined the impact of pasture vs. non-pasture-based feeding systems on the composition of the rumen metabolome with a series of metabolites highly associated with each production system [12]. However, little is known about the impact of varying pasture consumption levels on the rumen metabolome. Therefore, the objective of the present research was to characterise changes in the metabolite profile of bovine ruminal fluid samples, as a result of different pasture feeding levels following the consumption of pasture, PMR or TMR diets.

## 2. Materials and Methods

### 2.1. Ethical Approval

Consent for experimental procedures involving cows at Teagasc Animal and Grassland Research and Innovation Centre was authorised by the Health Products Regulatory Authority and approved by the Teagasc Animal Ethics Committee on 20 March 2020. The licence number received by the Health Products Regulatory Authority for this project is AE19132-P110.

### 2.2. Experimental Design

Rumen-cannulated cows (N = 9, Holstein-Friesian breed, average lactation number = 5, mean body weight = 550 kg) were assigned to three dietary regimes at the Teagasc Animal and Grassland Research and Innovation Centre, Moorepark, Fermoy, Co. Cork, Ireland. The diet treatments for this study were previously outlined, with Table 1 and Table 2 detailing the exact chemical composition of the ingredients involved in dietary regimes [18,19]. In brief, Group 1 (pasture) were maintained outdoors and consumed a diet consisting of perennial ryegrass (*Lolium perenne* L., 95% of fresh matter annual intake) through rotational grazing with supplemented concentrates (5% of fresh matter annual intake), which was provided during milking. Group 2 (TMR) were maintained indoors throughout the experiment and consumed a TMR feed consisting of 9 kg maize silage, 4.5 kg grass silage and 9 kg concentrates (40:20:40 dry matter basis), which was given ad libitum through electronically controlled Roughage Intake Control system feed bins (Hokofarm Group B.V., Marknesse, Netherlands), achieving ~10% refusal rate. Group 3 (PMR) consumed a partial mixed ration (PMR) diet, combining rotational grazing and supplemented concentrates, as per Group 1, between morning and evening milkings, and indoor feeding, as per Group 2, between the evening and morning milkings. Moreover, the PMR contained 4.5 kg of concentrate, 4.5 kg and 2.25 kg of maize and grass silage, respectively, and 9 kg of grazed grass, with the quantity of TMR consumed by the cows based off of the energy demands of the cows. The mixed ration feed was supplied to both group 2 and 3 at 08:30 h daily. Group 1 and 3 cows were kept outdoors in paddocks during the day, with the daily herbage allocation in the paddock based on the residency time in the paddock and number of cows within the group. The daily herbage allowance provided to groups 1 and 3 was approximately 17 kg DM (dry matter) for cow-1 day-1. Herbage mass was estimated twice weekly prior to grazing by group 1 and 3, by harvesting two strips in the area due to be grazed using an Etesia mower (Etesia UK Ltd., Warwick, UK). DM yield was calculated by drying a subsample at 90 °C for 15 h. In group 1, the target pre-grazing herbage mass was 1300–1500 kg DM ha^−1^ and 1000 to 1200 kg DM ha^−1^ for group 3, with a target post-grazing sward height of 4–4.5 cm for both groups [19]. Moreover, cows in group 1 and 3 were fed individually during milking, therefore maintaining post-grazing sward height and ensuring adequate feeding throughout the feeding trial.

Due to the COVID-19 lockdown from March to June 2020, rumen sampling took place between July 2020 and April 2021, with samples obtained from mid (July and August 2020), late (September and October 2020) and early (April and May 2021) lactation, similar to that described in previous research [3,12]. The mean days in milk were similar for all cows prior to starting treatment (125 in 2020 and 40 in 2021). Cows were rotated between each dietary regime in a Latin square design; therefore, each animal was rotated between each of the feeding systems during each stage of lactation (early, mid and late). Cows were allocated to the dietary regime for 16 days, with rumen samples obtained on two consecutive days (day 15 and 16) within early, mid and late lactation, at 07:00 h (morning) and 14:30 h (evening) following milking by the same person to ensure consistency. Following evening sampling on day 16, cows were rotated into a new dietary regime and given 14 days to acclimatise prior to sampling again on days 15 and 16. Like previous studies, rumen contents were obtained from various parts of the rumen (top, bottom, front and back) to ensure the samples was representative of the entire rumen content, and subsequently squeezed through three layers of synthetic cheese cloth, resulting in liquid and solid portions of rumen samples [12,20]. The resulting ruminal fluid samples were collected in sterile containers, snap-frozen using liquid nitrogen and stored at −80 °C until metabolomic analysis.

### 2.3. Metabolomic Analysis

Metabolomic analysis of the rumen fluid was preformed using a nuclear magnetic resonance (^1^H-NMR) (Varian Limited, Oxford, UK) spectroscopy approach. Rumen samples were defrosted for 2 h at room temperature. Following centrifugation at 2897× *g* for 15 min at 18 °C the rumen supernatant was removed. Briefly, the rumen extracts were further filtered (Whatman 0.22 μM syringe filters). The rumen filtrate (N = 309) was transferred to 1.5 mL Eppendorf tubes, followed by the addition of 50 μL deuterium oxide (D_2_O) (Sigma Aldrich, St. Louis, Missouri, USA) and 10 μL sodium trimethylsilyl [2,2,3,3-2H4] proprionate (TSP).

Spectra were acquired from a 600 MHz Varian Spectrometer (Varian Limited, Oxford, United Kingdom), with the first increment of a nuclear Overhauser enhancement spectroscopy pulse sequence, at 25 °C. Moreover, spectra were acquired using 16,384 complex data points with 256 scans, with water suppression achieved during a relaxation delay (3 s) and a 100 m/s mixing time. All ^1^H-NMR rumen spectra were referenced to TSP at 0.0 parts per million (ppm) and processed manually with the Chenomx NMR Suite (version 7.7) by using a line broadening of 0.2 Hz, followed by phase and baseline correction. The water region (4.5–5.0 PPM) was excluded from analysis. In total, 50 metabolites were identified and profiled based on the Chenomx 600 MHz Library and The Human Metabolome Database (HMBD).

### 2.4. Statistical Analysis

Prior to statistical analysis, datasets were normalised to the total sum of each sample. To assess the impact of dietary regime on the metabolite composition of the samples while controlling for lactation stage, general linear model analysis was applied to the morning (N = 153) and evening (N = 156) rumen ^1^H-NMR datasets with a false discovery rate (FDR) adjusted *p*-value ≤ 0.05 considered statistically significant using MetaboAnalyst 5.0 software (www.metaboanalyst.ca (accessed on 10 October 2023)). FDR correction was conducted using the Benjamani–Hochberg procedure. Following this, post hoc analysis to determine differences between the dietary regimes was conducted using Bonferroni correction, with an adjusted *p*-value < 0.05 considered statistically significant, performed using SPSS version 27.0 (IBM, Chicago, IL, USA). Pearsons correlations were determined between dietary regime and rumen metabolites at both morning and evening timepoints, controlling for lactation, again with an FDR-adjusted *p*-value ≤ 0.05 considered statistically significant, using Metaboanalyst 5.0. Multivariate statistical analysis on Group 1 (pasture) morning and evening ruminal fluid samples using SIMCA (SIMCA Version 13.0.3.0 Umetrics, AB). The dataset was scaled using pareto scaling. Principal component analysis (PCA) was conducted, providing an overview of the data. Partial least squares discriminant analysis (PLS-DA) was preformed to examine differences between the morning and evening grass-fed ruminal fluid samples. PLS-DA models were validated using permutation testing. Consequently, regularised canonical correlation analysis (rCCA) was performed to examine the relationship between morning and evening ruminal fluid metabolite profiles (N = 286) in R (version 4.1.3) using the mixOmics package (version 6.28.0). Consequently, a correlation network was built with a threshold of ≥0.15 using Cytoscape (version 3.10.1). Graphs were generated using Metaboanalyst (correlation coefficient graphs) (version 5.0), Microsoft Excel (bar graphs) (version 2407), Cytoscape (network) (version 3.10.2) and SIMCA (PCA and PLS-DA score plots) (version 13.0.3).

## 3. Results

### 3.1. Pasture Feeding Alters the Metabolite Profile of Bovine Ruminal Fluid

**Figure 1:**
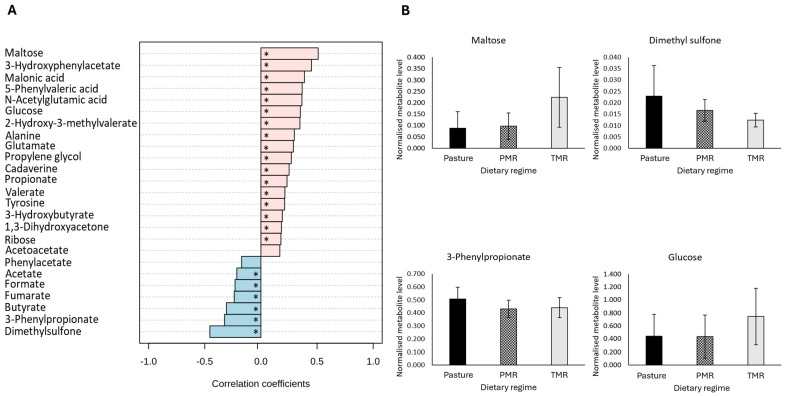
The impact of dietary regime on the metabolite composition of morning bovine ruminal fluid samples. (**A**) Pearsons correlation analysis between dietary regime and morning bovine ruminal fluid metabolites. Pearson correlations were controlled for lactation stage, with an FDR-adjusted *p*-value ≤ 0.05 considered statistically significant. (**B**) The subset of significantly different metabolites identified in morning bovine ruminal fluid samples. An analysis was performed using a general linear model analysis controlling for lactation stage to determine the significant metabolites across dietary regimes, pasture, PMR and TMR (FDR-adjusted *p*-value ≤ 0.05). Abbreviations are as follows: PMR, partial mixed ration; TMR, total mixed ration; FDR, false discovery rate; * denotes a statistically significant correlation (FDR-adjusted *p*-value ≤ 0.05).

Analysis of the data revealed an outlying sample, which was subsequently removed from the dataset. Data analysis was conducted on the remaining samples (N = 308). A detailed analysis of metabolite data revealed that a total of 29 metabolites were significantly different across dietary regimes in morning ruminal fluid (FDR-adjusted *p*-value ≤ 0.05) (Table 3). Multivariate pattern correlation analysis revealed that 21 metabolites in the morning sample set were significantly correlated with dietary regime (Figure 1A) (FDR-adjusted *p*-value ≤ 0.05). Positive correlations indicate an increase in metabolite levels in TMR-derived samples, whereas negative correlations indicate that the highest levels were in the pasture-derived samples. Metabolites with the highest levels in TMR-derived samples include the carbohydrates maltose and glucose, 3-hydroxyphenylacetate, N-acetylglutamic acid and 2-hydroxy-3-methylvalerate (Figure 1A,B) (FDR-adjusted *p*-value ≤ 0.05). Further analysis of the significantly different metabolites highlighted that the majority of the metabolites were different in the pasture-fed samples compared to the TMR/PMR groups, with only 29% of significantly different metabolites showing differences between the TMR and PMR dietary groups (FDR-adjusted *p*-value ≤ 0.05). The assessment of the morning ruminal fluid metabolite profile showed that the short-chain fatty acids acetate and butyrate were significantly higher in ruminal fluid from pasture-fed cows, whereas propionate was significantly lower in samples from pasture-based cows (Table 3) (FDR-adjusted *p*-value ≤ 0.05).

**Figure 2:**
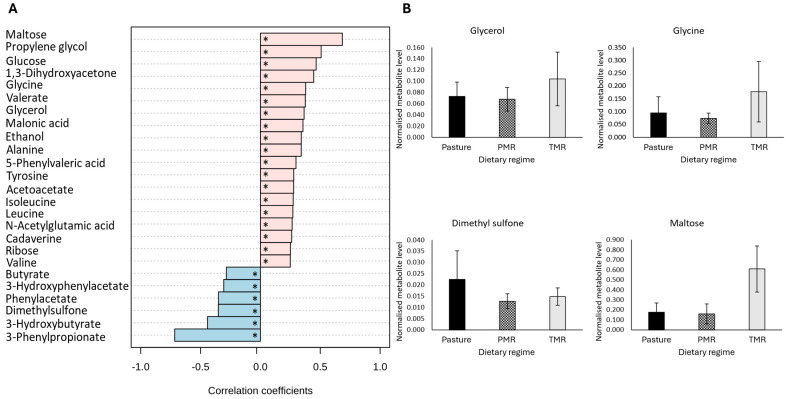
The impact of dietary regime on the metabolite composition of evening bovine ruminal fluid samples. (**A**) The Pearsons correlation analysis between dietary regime and morning bovine ruminal fluid metabolites. Pearson correlations were controlled for lactation stage, with an FDR-adjusted *p*-value ≤ 0.05 considered statistically significant. (**B**) The subset of significantly different metabolites identified in evening bovine ruminal fluid samples. Analysis performed using a general linear model analysis controlling for lactation stage to determine the significant metabolites across dietary regimes, pasture, PMR and TMR (FDR-adjusted *p*-value ≤ 0.05). Abbreviations are as follows: PMR, partial mixed ration; TMR, total mixed ration; FDR, false discovery rate; * denotes a statistically significant correlation (FDR-adjusted *p*-value ≤ 0.05).

Consistent with the morning samples, dietary regime had a significant impact on the evening ruminal fluid metabolome. A total 38 metabolites were significantly different across dietary regime (FDR-adjusted *p*-value ≤ 0.05) (Table 4). Similar to the morning samples, pattern correlation analysis revealed that 30 metabolites correlated significantly with dietary regime, including the sugar metabolites maltose, glucose and glycerol as well as the amino acid-related metabolites glycine, alanine and tyrosine, which were significantly correlated with the TMR diet (Figure 2A,B) (FDR-adjusted *p*-value ≤ 0.05). Overall, the results for the evening ruminal fluid samples corroborate those obtained for the morning samples, indicating the reproducibility of the data across sample sets, with acetate and butyrate significantly higher and the amino acid-related metabolites lysine, leucine and valine significantly lower in pasture-based samples (Table 4) (FDR-adjusted *p*-value ≤ 0.05). Interestingly, the differences between metabolites in the PMR and TMR dietary groups were more pronounced in the evening samples (Table 4) (FDR-adjusted *p*-value ≤ 0.05).

### 3.2. Relationship between Ruminal Fluid Morning and Evening Samples

**Figure 3:**
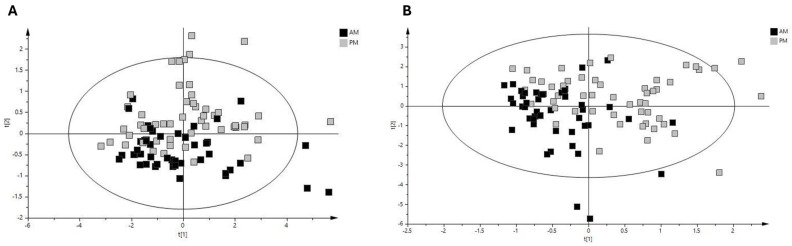
Diurnal variation in metabolite profile of pasture-based bovine ruminal fluid. (**A**) PCA score plot of ^1^H-NMR pasture group AM and PM samples (R^2^X = 0.982; Q^2^ = 0.839). (**B**) PLS-DA score plot of ^1^H-NMR pasture group AM and PM samples (R^2^X = 0.938; Q^2^ = 0.425). Black squares represent AM pasture group ruminal fluid samples and grey squares represent PM pasture group ruminal fluid samples. Abbreviations are as follows: ^1^H-NMR, proton nuclear magnetic resonance; PCA, principal component analysis; PLS-DA, partial least squares discriminant analysis; AM, morning bovine ruminal fluid samples; PM, evening bovine ruminal fluid samples; R^2^X, estimation of variance in data explained by model; Q^2^, estimation of predictive ability explained by model.

**Figure 4:**
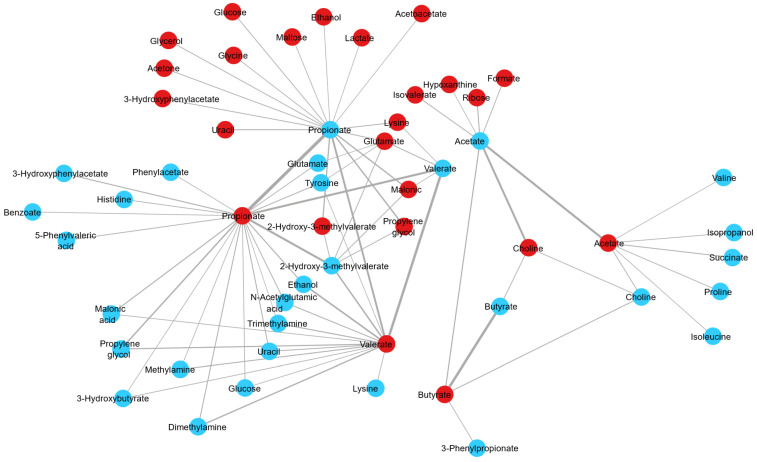
A network graph depicting the positive correlations derived from regularised canonical correlation analysis between AM (N = 144) and PM (N = 144) bovine ruminal fluid sample metabolites, with a correlation coefficient > 0.15. The edge is sized according to the correlation strength, with a wider edge indicating a higher correlation. Blue circles represent AM bovine ruminal fluid metabolites and red circles present PM bovine ruminal fluid metabolites. Abbreviations are as follows: AM, morning bovine ruminal fluid metabolites; PM, evening bovine ruminal fluid metabolites.

The assessment of fluctuations occurring in the ruminal fluid metabolomic profile across the day was performed using samples from the morning and evening. To explore further the differences due to sample time, PCA of the samples from pasture-fed cows was performed, with a clear separation of samples based on sampling time evident (R^2^X value 0.982, Figure 3A). A robust PLS-DA model was built (Q^2^ value 0.425, R^2^X value 0.938, Figure 3B) with clear separation between morning and evening samples. Permutation testing determined that the model was robust (intercepts R^2^X = (0.0, 0.0593), Q^2^ = (0.0, −0.146)). Metabolites identified as discriminant between morning and evening pasture samples included the short-chain fatty acids acetate, butyrate, propionate, 3-phenylpropionate and glucose, with acetate levels decreasing and butyrate, propionate, 3-phenylpropionate and glucose levels increasing from morning to evening sampling in cows fed a pasture-based diet (Table 3 and Table 4). To further explore the relationship between morning and evening ruminal fluid samples, we examined the correlation between the metabolite profiles of all samples. Paired analysis (morning and evening samples) of the ruminal fluid samples revealed that metabolites such as acetate, aspartate, butyrate, choline, glutamate, propionate, valerate and 2-hydroxy-3-methylvalerate were positively correlated between morning and evening samples (Figure 4) (correlation coefficient ≥ 0.15). Furthermore, network analysis illustrated that metabolites such as 2-hydroxy-3-methylvalerate, acetate, butyrate, ethanol, propylene glycol, valerate and propionate in morning ruminal fluid samples were positively correlated with multiple metabolites in evening samples such as the short-chain fatty acids acetate, butyrate, propionate, valerate and vitamins and alcohols such as choline and propylene glycol (Figure 4). In summary, the results of this analysis demonstrate the complex relationship between various metabolites in bovine ruminal fluid across the day.

## 4. Discussion

The present findings demonstrate the significant impact of a pasture-based diet on the ruminal fluid metabolome, with marked differences occurring in metabolite classes such as short-chain fatty acids and carbohydrate-related metabolites. While some metabolites, including dimethylsuflone, and the amino acids glutamate and isoleucine, were significantly different across all dietary regimes, the majority of metabolite differences were between the pasture-based feeding regime and the PMR and TMR diets. Understanding these alterations is imperative for the dairy industry in order to optimise animal nutrition, improving feed efficiency and milk productivity.

In our study, 3-phenylpropionate was significantly higher in pasture-derived ruminal fluid compared to the TMR group. This was in accordance with previous studies, where the concentration of 3-phenylpropionate decreased following an increase in the proportion of dietary barley grain [13]. Moreover, the consumption of grass silage resulted in significantly higher levels of 3-phenylpropionate in rumen samples when compared to a red clover dietary regime [11]. It is observed that increasing ruminal pH, which is associated with higher forage diets, leads to an increase in 3-phenylpropionate concentrations [14,21]. In agreement with this, the analysis of ruminal fluid from cows fed a diet consisting of 80% forage and 20% concentrates versus a high-concentrate diet found 3-phenylpropionate was significantly lower in high-concentrate rumen samples [10]. Interestingly, lower 3-phenylpropionate concentrations have been correlated with higher ruminal glucose levels [21]. Our results identified an increase in carbohydrate compounds in the TMR-derived rumen samples, with glucose, maltose and ribose found in significantly higher levels. Attributed to the increased consumption of fermentable carbohydrates through higher starch containing feeds, many studies have found that rumen samples from cows fed a TMR/high-concentrate diet contained higher levels of both D-maltose and glucose [12,13,16]. For example, increasing the starch contribution within the diet from 30 to 45% grain increases maltose levels, altering both starch and sucrose metabolism in the rumen [13,14]. Furthermore, increased ribose levels occur as a result of alterations to purine and pyrimidine pathways and amino acid metabolism [13,14]. Interestingly, our results indicated significantly higher levels of dimethyl sulfone in pasture-fed ruminal fluid samples. It has been reported that dimethyl sulfone is the metabolite most responsible for separation between pasture and concentrate profiles [12]. It is formed through the catabolism of methionine in the rumen, with methionine found in high quantities in grass feed systems [22,23]. Overall, the examination of the ruminal fluid metabolite profiles in this study highlighted the influence of concentrate-based dietary regimes on sugar-based metabolites, resulting in higher glucose, maltose, ribose and glycerol levels attributed to increased starch consumption.

The current study reported a significant increase in biogenic amines such as cadaverine in TMR-derived samples. These results are consistent with previous research where cadaverine levels were higher following an increase in grain consumption [13]. Moreover, increasing the proportion of concentrates within the diet results in higher biogenic amine levels leading to rumen acidification and unfavourable alterations of the rumen metabolome [13,14]. Furthermore, in our study, a number of amino acids including the branched-chain amino acids, alanine and tyrosine, had higher levels in TMR-derived ruminal fluid samples. Consistent with previous research, increasing the proportion of grain increased the levels of alanine, lysine, leucine and valine within the rumen [13]. Collectively, our analysis corroborates the findings of previous research, and further highlights the impacts of a TMR diet on amino acid and biogenic amine compound classes.

Similar to previous analysis, our study showed that the short-chain fatty acids, propionate, acetate and butyrate, are the metabolites most abundant in ruminal fluid samples [4,8,12,24]. In our study, perturbations in the levels of various short-chain fatty acids occurred within the rumen metabolome following the consumption of a pasture or concentrate dietary regime. The levels of acetate and butyrate were significantly higher in ruminal fluid samples from pasture-fed cows and propionate in samples from TMR-fed cows. Short-chain fatty acids are produced through fermentation in the rumen, acting as the principal energy source for cows supplying approximately 70% of their energy requirements [6,25]. In line with our results, numerous studies have identified higher ruminal propionate levels following an increased proportion of barley/concentrate within the diet [3,6,13,24]. Alterations occurring in propionate production within the rumen have consequential effects on both milk yield and quality, with high propionate levels associated with increased milk yield and decreased protein levels [25,26]. Acetate is responsible for producing approximately 50% of the total energy in the rumen, playing a regulatory role in milk fat synthesis within the mammary gland [27]. The fat content of milk is of critical importance to the dairy industry, with previous research showing that increasing the acetate to propionate ratio in the rumen had a positive association with milk fat levels, highlighting how a pasture-based regime may be beneficial in terms of milk composition [14,28]. However, alterations in the levels of compounds including acetic, propionate and butyric acid result in negative changes to methane emissions [29,30,31]. Methane production is influenced by feed type and fermentation rate, with recent studies demonstrating that feeding grass silage resulted in increased levels of acetate and butyrate, which are associated with higher methane emissions [11,32]. The synthesis of metabolites such as acetate and butyrate results in increased hydrogen levels, allowing for increased production of methane in the hydrogenotrophic pathway of the rumen [21]. Collectively, our results demonstrate the complex nature of the ruminal fluid metabolite role, and the crucial role which a pasture- or concentrate-based diet may play for both milk production and quality.

In agreement with previous studies, our results highlight distinct diurnal fluctuations occurring in the rumen metabolome across the day [32]. The levels of metabolites such as propionate, lactate and ethanol were higher in evening samples compared to morning samples. Previous work has attributed this increase to rumen fermentation processes following feed intake [33]. As demonstrated in our results, although highly correlated, there were distinct differences between the morning and evening pasture-based ruminal fluid metabolites such as acetate, propionate and butyrate. In our study, levels of butyrate and propionate were higher in evening rumen samples compared to morning samples. In accordance with our results, many studies have identified higher levels of short-chain fatty acids such as acetate, propionate and butyrate in evening rumen samples following feeding [3,32]. While these studies did not analyse the same metabolites as those examined here, similar patterns with respect to ruminal fluctuations occur. Understanding the modulations which occur in the rumen following feeding and throughout the day are crucial to understanding the production of various metabolites and their association with methane emissions [33]. The differences found in our analysis highlight the importance of timing for sample collection and the need for clear guidance on the timeframe of collection of rumen samples.

## 5. Conclusions

In conclusion, the results of this study offer in-depth analysis into the impact of dietary regime on the metabolomic profile of the rumen and provide new insights into the impact of the differing of pasture levels. The findings of this study identify a strong diurnal impact on the metabolite profile of samples, highlighting the impact of both feeding time, dietary regime and sampling time on the rumen metabolome. There is a paucity of information on the impact of diet on the rumen metabolite profile, and our results contribute to existing literature on pasture versus concentrate dietary regimes, highlighting how rumen metabolite classes including carbohydrates, short-chain fatty acids and amino acids are affected. Therefore, future work should link changes in the rumen metabolome to the rumen microbiome and milk metabolome as understanding how modulation occurring in the metabolite profile of bovine ruminal fluid is of critical importance for the agri-food sector, due to its effects on the health status of the animal, animal productivity and composition of dairy products.

## Figures and Tables

**Table 1:** The average chemical composition of the TMR and PMR diet offered over the experimental period.

	Maize Silage	Grass Silage	Conc.	Parlour Conc.	Total TMR	Total PMR
DM (%)	33.70	30.71	93.87	89.00	58.11	41.54
CP (g/kg DM)	76.54	152.05	220.33	133.33	146.54	177.66
NDF (g/kg DM)	427.29	459.71	238.86	308.80	358.43	363.69
Ash (g/kg DM)	35.65	93.92	71.68	94.53	63.36	74.59
Starch (%)	31.19	N/A	N/A	N/A	12.06	6.59
OM Digestibility (g/kg DM)	760.17	739.00	849.00	840.6	792.90	815.66
Gross energy (MJ/kg DM)	19.22	18.64	19.57	18.49	19.19	19.20

Values are presented as the mean for each ingredient and the total of the TMR and PMR diets. Abbreviations are as follows: %, percentage; g/kg DM, grammes per kilogramme of dry matter; PMR, partial mixed ration; TMR, total mixed ration; N/A, not applicable; MJ/kg DM, megajoules per kilogramme; Conc., concentrate; DM, dry matter; CP, crude protein; NDF, neutral detergent fibre; OM, organic matter.

**Table 2:** The average chemical composition of the herbage grazed by Pasture and PMR cows.

	Pasture	PMR
DM (%)	208.31	221.18
CP (g/kg DM)	845.13	843.23
NDF (g/kg DM)	366.99	374.57
Ash (g/kg DM)	208.42	218.67
Starch (%)	85.39	87.64
OM Digestibility (g/kg DM)	19.22	19.26
Gross energy (MJ/kg DM)	208.31	221.18

Values are presented as the mean for each component of the GRASS and PMR diets. Abbreviations are as follows: %, percentage; g/kg DM, grammes per kilogramme of dry matter; PMR, partial mixed ration; MJ/kg DM, megajoules per kilogramme; DM, dry matter; CP, crude protein; NDF, neutral detergent fibre; OM, organic matter.

**Table 3:** Average metabolite levels (%) in morning bovine ruminal fluid samples in each dietary regime.

Metabolite AM	Pasture		PMR		TMR		FDR
1,3-Dihydroxyacetone	0.03	(0.02)	0.03	(0.01)	0.03	(0.01)	<0.05
2-Hydroxy-3-methylvalerate	0.07	(0.02) ^a^	0.09	(0.03) ^b^	0.09	(0.03) ^b^	<0.001
3-Hydroxybutyrate	0.03	(0.01) ^ab^	0.03	(0.01) ^a^	0.03	(0.01) ^b^	<0.05
3-Hydroxyphenylacetate	0.02	(0.01) ^a^	0.03	(0.01) ^b^	0.03	(0.01) ^b^	<0.001
3-Phenylpropionate	0.51	(0.09) ^a^	0.43	(0.07) ^b^	0.44	(0.08) ^b^	<0.001
5-Phenylvaleric acid	0.18	(0.09) ^a^	0.20	(0.08) ^ab^	0.23	(0.08) ^b^	<0.001
Acetate	66.37	(3.05) ^a^	62.90	(5.82) ^b^	64.18	(3.48) ^b^	<0.001
Acetoacetate	0.03	(0.01)	0.03	(0.01)	0.03	(0.01)	0.0.095
Acetone	0.03	(0.04)	0.03	(0.02)	0.04	(0.03)	0.610
Alanine	0.17	(0.06) ^a^	0.19	(0.04) ^ab^	0.20	(0.05) ^b^	<0.01
Aspartate	0.10	(0.05) ^a^	0.12	(0.04) ^b^	0.11	(0.04) ^ab^	<0.01
Benzoate	0.04	(0.03)	0.03	(0.01)	0.04	(0.02)	<0.05
Butyrate	10.50	(1.10) ^a^	8.99	(1.19) ^b^	9.41	(1.84) ^b^	<0.001
Cadaverine	0.05	(0.02) ^a^	0.05	(0.02) ^ab^	0.06	(0.02) ^b^	<0.01
Choline	0.02	(0.01) ^a^	0.01	(0.00) ^b^	0.02	(0.01) ^ab^	<0.05
Dimethyl sulfone	0.02	(0.01) ^a^	0.02	(0.00) ^b^	0.01	(0.00) ^c^	<0.001
Dimethylamine	0.01	(0.01)	0.01	(0.01)	0.01	(0.01)	0.498
Ethanol	0.10	(0.08)	0.12	(0.09)	0.10	(0.04)	0.433
Formate	0.09	(0.03) ^a^	0.09	(0.02) ^ab^	0.07	(0.02) ^b^	<0.01
Fumarate	0.01	(0.01) ^a^	0.01	(0.00) ^ab^	0.01	(0.00) ^b^	<0.05
Glucose	0.44	(0.34) ^a^	0.44	(0.33) ^ab^	0.75	(0.44) ^b^	<0.001
Glutamate	0.26	(0.07) ^a^	0.43	(0.13) ^b^	0.35	(0.14) ^c^	<0.001
Glycerol	0.06	(0.04)	0.06	(0.02)	0.06	(0.02)	0.216
Glycine	0.06	(0.02)	0.07	(0.02)	0.07	(0.02)	0.181
Histidine	0.02	(0.01)	0.02	(0.01)	0.02	(0.01)	0.528
Hypoxanthine	0.07	(0.03)	0.06	(0.02)	0.07	(0.03)	0.139
Imidazole	0.03	(0.02)	0.03	(0.01)	0.03	(0.01)	0.098
Isobutyrate	0.96	(0.20) ^a^	0.86	(0.09) ^b^	0.98	(0.17) ^ab^	<0.001
Isoleucine	0.04	(0.02)	0.04	(0.01)	0.04	(0.01)	0.056
Isopropanol	0.03	(0.01) ^a^	0.05	(0.03) ^b^	0.04	(0.02) ^ab^	<0.001
Isovalerate	0.61	(0.17) ^a^	0.54	(0.08) ^b^	0.57	(0.11) ^ab^	<0.05
Lactate	0.07	(0.07)	0.07	(0.03)	0.07	(0.02)	0.941
Leucine	0.05	(0.02)	0.05	(0.01)	0.05	(0.01)	0.289
Lysine	0.07	(0.03)	0.07	(0.03)	0.08	(0.02)	0.941
Malonic acid	0.02	(0.01) ^a^	0.02	(0.01) ^ab^	0.02	(0.01) ^b^	<0.001
Maltose	0.09	(0.07) ^a^	0.10	(0.06) ^a^	0.22	(0.13) ^b^	<0.001
Methanol	0.01	(0.02)	0.01	(0.01)	0.01	(0.01)	0.611
Methylamine	0.03	(0.03)	0.02	(0.03)	0.02	(0.02)	0.277
N-Acetylglutamic acid	0.20	(0.08) ^a^	0.23	(0.08) ^a^	0.27	(0.07) ^b^	<0.001
Phenylacetate	0.25	(0.14)	0.20	(0.09)	0.20	(0.11)	0.052
Proline	0.12	(0.07)	0.12	(0.04)	0.12	(0.04)	0.871
Propionate	16.10	(2.22) ^a^	20.23	(4.26) ^b^	18.19	(3.30) ^c^	<0.001
Propylene glycol	0.03	(0.02) ^a^	0.04	(0.03) ^ab^	0.04	(0.03) ^b^	<0.01
Ribose	0.24	(0.15) ^ab^	0.22	(0.10) ^a^	0.29	(0.12) ^b^	<0.05
Succinate	0.05	(0.04) ^ab^	0.05	(0.03) ^a^	0.04	(0.03) ^b^	0.064
Trimethylamine	0.03	(0.04) ^a^	0.03	(0.05) ^ab^	0.02	(0.02) ^b^	0.181
Tyrosine	0.04	(0.02)	0.05	(0.02)	0.05	(0.01)	<0.05
Uracil	0.07	(0.03)	0.07	(0.03)	0.08	(0.03)	0.155
Valerate	1.48	(0.76) ^a^	2.38	(1.50) ^b^	2.06	(0.82) ^b^	<0.001
Valine	0.06	(0.02)	0.06	(0.02)	0.06	(0.02)	0.575

Values are presented as the mean (SD). An FDR-adjusted *p*-value ≤ 0.05 was considered significant. ^a, b, c^: different superscript letters denote significant differences between dietary regimes analysed using general linear model controlling for the lactation stage. Abbreviations are as follows:; SD, standard deviation; PMR, partial mixed ration; TMR, total mixed ration; false discovery rate, FDR.

**Table 4:** Average metabolite levels (%) in evening bovine ruminal fluid samples in each dietary regime.

Metabolite AM	Pasture		PMR		TMR		FDR
1,3-Dihydroxyacetone	0.02	(0.01) ^a^	0.03	(0.01) ^b^	0.03	(0.01) ^b^	<0.001
2-Hydroxy-3-methylvalerate	0.09	(0.02) ^a^	0.10	(0.03) ^ab^	0.10	(0.04) ^b^	<0.05
3-Hydroxybutyrate	0.05	(0.02) ^a^	0.05	(0.01) ^b^	0.04	(0.01) ^c^	<0.001
3-Hydroxyphenylacetate	0.06	(0.04) ^a^	0.06	(0.02) ^a^	0.03	(0.02) ^b^	<0.001
3-Phenylpropionate	0.83	(0.20) ^a^	0.85	(0.13) ^a^	0.37	(0.11) ^b^	<0.001
5-Phenylvaleric acid	0.22	(0.09) ^a^	0.22	(0.08) ^a^	0.27	(0.11) ^b^	<0.001
Acetate	61.04	(4.46) ^a^	61.11	(3.20) ^a^	58.75	(3.92) ^b^	<0.01
Acetoacetate	0.03	(0.01) ^a^	0.03	(0.01) ^a^	0.04	(0.02) ^b^	<0.001
Acetone	0.07	(0.08) ^a^	0.04	(0.02) ^b^	0.09	(0.09) ^a^	<0.001
Alanine	0.18	(0.06) ^a^	0.20	(0.06) ^a^	0.32	(0.27) ^b^	<0.001
Aspartate	0.10	(0.04)	0.10	(0.04)	0.10	(0.05)	0.225
Benzoate	0.06	(0.02) ^ab^	0.07	(0.08) ^a^	0.04	(0.02) ^b^	<0.05
Butyrate	11.53	(1.47) ^a^	10.01	(2.13) ^b^	10.27	(1.96) ^b^	<0.001
Cadaverine	0.07	(0.04) ^a^	0.07	(0.02) ^a^	0.10	(0.03) ^b^	<0.001
Choline	0.02	(0.01)	0.02	(0.01)	0.02	(0.01)	0.074
Dimethyl sulfone	0.02	(0.01) ^a^	0.01	(0.00) ^b^	0.02	(0.00) ^b^	<0.001
Dimethylamine	0.03	(0.02)	0.03	(0.03)	0.03	(0.03)	0.385
Ethanol	0.32	(0.41) ^a^	0.22	(0.21) ^a^	0.93	(1.05) ^b^	<0.001
Formate	0.06	(0.02) ^ab^	0.06	(0.02) ^a^	0.05	(0.02) ^b^	<0.001
Fumarate	0.01	(0.01)	0.01	(0.00)	0.01	(0.01)	0.381
Glucose	0.88	(0.46) ^a^	0.66	(0.32) ^a^	1.61	(0.70) ^b^	<0.001
Glutamate	0.20	(0.05) ^a^	0.27	(0.09) ^b^	0.25	(0.10) ^b^	<0.001
Glycerol	0.07	(0.03) ^a^	0.07	(0.02) ^a^	0.10	(0.05) ^b^	<0.001
Glycine	0.10	(0.06) ^a^	0.07	(0.02) ^a^	0.18	(0.12) ^b^	<0.001
Histidine	0.03	(0.01)	0.02	(0.01)	0.02	(0.01)	0.257
Hypoxanthine	0.09	(0.03) ^ab^	0.08	(0.03) ^a^	0.10	(0.03) ^b^	<0.01
Imidazole	0.04	(0.02)	0.04	(0.02)	0.04	(0.02)	0.257
Isobutyrate	0.87	(0.21)	0.87	(0.17)	0.89	(0.14)	0.712
Isoleucine	0.06	(0.02) ^a^	0.04	(0.01) ^b^	0.08	(0.03) ^c^	<0.001
Isopropanol	0.05	(0.02) ^ab^	0.04	(0.01) ^a^	0.06	(0.03) ^b^	<0.01
Isovalerate	0.57	(0.16)	0.54	(0.11)	0.54	(0.12)	0.434
Lactate	0.08	(0.03)	0.07	(0.02)	0.08	(0.04)	0.113
Leucine	0.05	(0.02) ^a^	0.06	(0.02) ^a^	0.07	(0.04) ^b^	<0.01
Lysine	0.10	(0.05) ^a^	0.08	(0.04) ^a^	0.13	(0.07) ^b^	<0.001
Malonic acid	0.02	(0.01) ^a^	0.02	(0.01) ^a^	0.03	(0.01) ^b^	<0.001
Maltose	0.18	(0.09) ^a^	0.16	(0.10) ^a^	0.61	(0.23) ^b^	<0.001
Methanol	0.02	(0.02) ^a^	0.02	(0.01) ^a^	0.03	(0.03) ^b^	<0.01
Methylamine	0.17	(0.11) ^ab^	0.19	(0.14) ^a^	0.13	(0.08) ^b^	<0.05
N-Acetylglutamic acid	0.26	(0.08) ^a^	0.27	(0.07) ^a^	0.31	(0.05) ^b^	<0.01
Phenylacetate	0.32	(0.18) ^a^	0.28	(0.11) ^a^	0.21	(0.08) ^b^	<0.001
Proline	0.11	(0.05)	0.11	(0.04)	0.12	(0.05)	0.090
Propionate	18.43	(2.87) ^a^	19.87	(2.09) ^b^	19.24	(3.42)	<0.05
Propylene glycol	0.04	(0.02) ^a^	0.04	(0.01) ^a^	0.10	(0.06) ^b^	<0.001
Ribose	0.27	(0.11) ^a^	0.28	(0.09) ^a^	0.34	(0.13) ^b^	<0.01
Succinate	0.02	(0.02)	0.02	(0.01)	0.01	(0.01)	0.101
Trimethylamine	0.11	(0.14)	0.06	(0.06)	0.10	(0.13)	0.080
Tyrosine	0.05	(0.02) ^a^	0.05	(0.02) ^a^	0.06	(0.02) ^b^	<0.001
Uracil	0.10	(0.03)	0.09	(0.03)	0.11	(0.04)	<0.05
Valerate	1.88	(0.65) ^a^	2.30	(1.10) ^a^	2.77	(1.00) ^b^	<0.001
Valine	0.06	(0.02) ^a^	0.06	(0.02) ^a^	0.09	(0.08) ^b^	<0.01

Values are presented as mean (SD). FDR-adjusted *p*-value ≤ 0.05 was considered significant. ^a, b, c^: different superscript letters denote significant differences between dietary regimes analysed using general linear model controlling for lactation stage. Abbreviations are as follows: N, number of samples; SD, standard deviation; PMR, partial mixed ration; TMR, total mixed ration; false discovery rate, FDR.

## Data Availability

The raw data supporting the conclusions of this article will be made available by the authors on request.
